# Circular RNA circ_0008274 upregulates granulin to promote the progression of hepatocellular carcinoma via sponging microRNA -140-3p

**DOI:** 10.1080/21655979.2021.1926195

**Published:** 2021-05-18

**Authors:** Chanchan Gao, Yazhou Wen, Feng Jiang, Xuyu Gu, Xinhua Zhu

**Affiliations:** a Department of Oncology, Zhongda Hospital Affiliated to Southeast University, Nanjing, China; b Department of Anesthesiology, Zhongda Hospital Affiliated to Southeast University, Nanjing, China; c Department of General Surgery, Nanjing Drum Tower Hospital, Nanjing, China

**Keywords:** HCC, circRNA, microRNA, granulin

## Abstract

Circular RNA (circRNA) features prominently in the progression of hepatocellular carcinoma (HCC), of which the biological function and potential mechanism of circ_0008274 in HCC are obscure. The present study aims to explore circ_ 0008274’s biological functions and underlying mechanisms in HCC. The expressions of circ_0008274, miR-140-3p and Granulin (GRN) mRNA in HCC tissues and cells were investigated by quantitative real-time polymerase chain reaction. Besides, GRN protein expression was measured by Western blot. Furthermore, chi-square test was used to probe the interrelation between circ_0008274 expression and clinicopathological parameters. In addition, cell counting kit-8 (CCK-8) and EdU assays were applied to detect cell proliferation. Moreover, transwell assay was used to detect cell migration and invasion. What’s more, bioinformatics prediction, dual-luciferase reporter gene assay and RNA Immunoprecipitation experiments were used to corroborate the targeting interrelations among circ_0008274, miR-140-3p and GRN. Herein we reported that circ_0008274 was highly expressed in HCC, and its high expression enhanced the proliferation, migration, and invasion of HCC cells, while depleting circ_0008274 inhibited the malignant biological behaviors of HCC cells. Mechanistically, circ_0008274 upregulates GRN expressions via adsorbing miR-140-3p to expedite the progression of HCC.

## GRAPHICAL ABSTRACT

**Figure:**
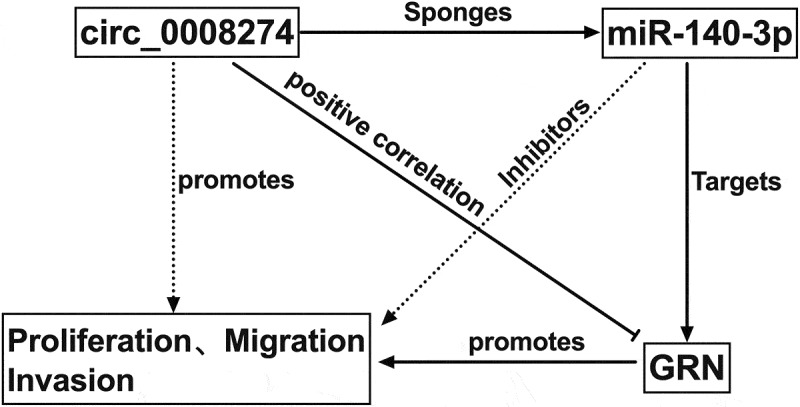

## Introduction

Hepatocellular carcinoma (HCC) is one of the major cancers that endanger human health worldwide, and its incidence is increasing gradually [1]. In recent years, despite the continuous progress in surgical resection, transplantation, radiotherapy and chemotherapy, the 5-year survival rate of HCC patients is still low ensuing from their high local invasion ability and distant metastasis rate [2]. It is therefore important to decipher the mechanism of HCC pathogenesis and explore new treatment targets.

**Figure 1.**
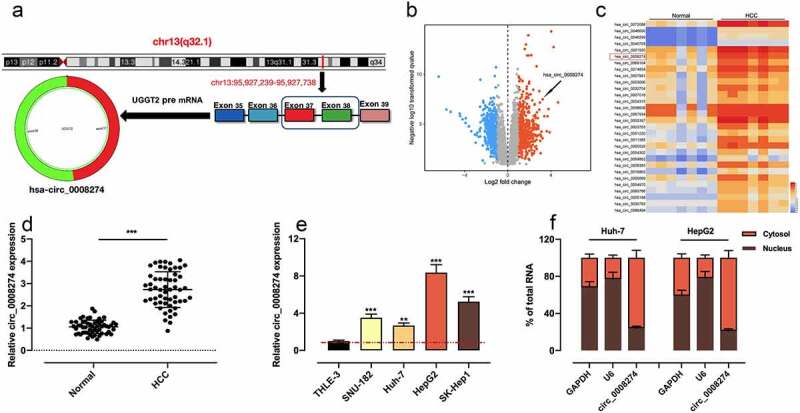
Circ_0008274 expression is significantly up-regulated in HCC tissues and cells

Circular RNA (circRNA) is mainly produced by precursor mRNA back-splicing of the 3 ‘and 5ʹ ends of exons, with the characteristics of stable structure and conserved sequence [3,4]. A large number of circRNAs have been discovered, and they are reported to participate in regulating the progression of HCC [5]. For example, circ104718, as a competing endogenousRNA (ceRNA), accelerates the progression of HCC via modulating the miR-218-5p/TXNDC5 axis [6]. CircMAT2B is in significantly high expression in HCC, and promotes HCC progression by modulating miR-338-3p/PKM2 axis under hypoxic condition [7]. Notably, it has been reported that circ_0008274 is up-regulated in papillary thyroid carcinoma tissues, and that the highly expressed circ_0008274 is relevant to poor prognosis in patients [8]. In this study, by analyzing the dataset from Gene Expression Omnibus (GEO), we demonstrated that circ_0008274 was in high expression in HCC tissues; and circ_0008274 was generated from the transcript of UDP-glucose glycoprotein glucosyltransferase 2 (UGGT2) (Figure 1(a)). Considering UGGT2 is an important regulator in HCC progression [9], we hypothesized that circ_0008274 may also participate in HCC pathogenesis.

A. Circ_0008274 was generated from the transcript of UGGT2 gene.B. Volcanic map was used to show the changes of circRNAs’ expression in HCC tissues compared with normal tissues adjacent to cancer. CircRNAs whose expression was significantly up-regulated were marked with orange, while those with significantly down-regulated expression were marked with blue (log_10_|fold change| > 1, *P* < 0.05).C. The differentially expressed circRNAs in HCC tissues and adjacent normal tissues were shown in the heatmap.D. The expression of circ_0008274 in HCC tissues and adjacent tissues was detected by qRT-PCR.E. The expression of circ_0008274 in HCC cell lines and THLE-3 cell was detected by qRT-PCR.F. The subcellular localization of circ_0008274 in Huh-7 and HepG2 cells was detected by qRT-PCR.** *P* < 0.01, and *** *P* < 0.001.

MicroRNAs (miRNAs) are single-stranded small-molecule RNAs with 18–27*nt*. They are devoid of an open reading frame and the ability to encode protein, and exert their biological functions via modulating the target genes’ expression [10]. The abnormal expression of miRNA is involved in cancer progression [11]. For example, miR-140-3p can inhibit the progression of HCC via targeting granulin (GRN) and restraining the MAPK signaling pathway [12]. However, the upstream regulatory mechanism of miR-140-3p/GRN axis in HCC is unclear.

Bioinformatics analysis shows that circ_0008274 can target miR-140-3p/GRN axis. In this work, we hypothesized that circ_0008274 promotes the occurrence and development of HCC via regulating miR-140-3p/GRN axis. The experiments were performed to validate the scientific hypothesis mentioned above.

## Materials and methods

### Ethical statement and tissue samples

The tissues adjacent to cancer and cancer tissues of 55 patients with HCC diagnosed in Zhongda Hospital from May 2018 to June 2020 were collected. After removal, all HCC samples were immediately frozen in liquid nitrogen and subsequently stored until the follow-up analysis. All the cancer tissues samples were histologically confirmed to be HCC. None of the patients included received chemotherapy, radiotherapy and targeted therapy before the surgery. The patients’ informed consent was obtained, and the collection and use of human tissues was endorsed by the Ethics Committee of Zhongda Hospital (Approval no: 2016–08).

### Cell culture and transfection

Human HCC cell lines SNU-182 and SK-Hep1, human embryonic kidney cells (HEK293T) and normal liver cells THLE-3 were available from American Type Culture Collection (ATCC, Manassas, VA, USA). Human HCC cell line Huh-7 and HepG2 were available from Type Culture Collection of Chinese Academy of Sciences (Shanghai, China). The cells mentioned above were routinely cultured in DMEM (Thermo Fisher Scientific Inc., Rockford, IL, USA) containing 10% fetal bovine serum (FBS, Shanghai Biyuntian Biotechnology Co., Ltd, Shanghai, China), 100 U/mL penicillin and 0.1 mg/mL streptomycin (Shanghai Biyuntian Biotechnology Co., Ltd, Shanghai, China) at 37°C in 5% CO_2_. Next, the medium was accordingly replaced with fresh medium every 2 ~ 3 days. Besides, cells were subcultured with 0.25% trypsin (Roche, Basel, Swizerland) upon reaching 70–80% confluence. Furthermore, circ_0008274 overexpression plasmid (pcDNA-circ_0008274), empty vector (pcDNA-NC), small interfering RNA (siRNA) oligonucleotides targeting circ_0008274 (si-circ_0008274-1, si-circ_0008274-2), siRNA oligonucleotides targeting human GRN (si-GRN), siRNA negative control (si-NC), miR-140-3p mimics and its control (miR-NC), miR-140-3p inhibitors and its control (Inh-NC) were all constructed by GenePharma (Shanghai, China). Above vectors and oligonucleotides were accordingly transfected with lipofectamine 3000 (Invitrogen, Carlsbad, CA, USA) into Huh-7 or HepG2 cells. 24 h later, quantitative real-time polymerase chain reaction (qRT-PCR) was adopted to examine the transfection efficiency.

### qRT-PCR

The total RNA was extracted from tissues and cells by TRIzol Regent (Invitrogen, Carlsbad, CA, USA), with its RNA concentration and purity detected. Next, the total RNA was reversely transcribed by First Strand cDNA Synthesis Kit (Thermo Fisher Scientific Inc., Rockford, IL, USA) and Mir-X™miRNA First-Strand Synthesis kit (Clontech, Shanghai, China), respectively. With the cDNA as the template, qRT-PCR was subsequently performed with SYBR®Premix-Ex-Taq™ (Takara, Tokyo, Japan) on ABI7300 system (Thermo Fisher Scientific Inc., Rockford, IL, USA). GAPDH and U6 were used as the internal control for mRNA and miRNA, respectively. The specific primer sequences are detailed in Table 1. To detect the subcellular location of circ_0008274 in HCC cells, the nuclear and cytoplasmic RNA of Huh-7 and HepG2 cells were isolated by PARIS™ kit (Thermo Fisher Scientific Inc., Rockford, IL, USA), respectively. Next, circ_0008274 expression in cytoplasm and nucleus of the cells was examined by qRT-PCR, with GAPDH and U6 as the controls, respectively.

**Table 1.** Primers used for qRT-PCR

	Forward	Reverse
circ_0008273	5ʹ-CCAGATCTCCCTGTTTCACC CCTGTCCTCCTAAACCTCCAAG-3’	5ʹ-CTTGCCCTCTTTGGCTCTCT-3’
miR-140-3p	5ʹ-TACCACAGGGTAGAACCACGG-3ʹ	5ʹ-CCACAGGGTAGAACCACGG-3ʹ
GRN	5ʹ-ATCTTTACCGTCTCAGGGACTT-3’	5ʹ-CCATCGACCATAACACAGCAC-3’
U6	5ʹ-TGCGGGTGCTCGCTTCGGCAGC-3’	5ʹ-CCAGTGCAGGGTCCGAGGT-3’
GAPDH	5ʹ-GCACCGTCAAGGCTGAGAAC-3’	5ʹ-TGGTGAAGACGCCAGTGGA-3’

### Cell Counting Kit-8 assay (CCK-8)

Huh-7 and HepG2 cells were trypsinized, then the cell density was adjusted to 2 × 10^4^ cells/mL. Subsequently, the cells were cultured in 96-well plates with 100 μL cell suspension per well. At 24, 48, 72 and 96 h, the cells were, respectively, incubated with 10 μL of CCK-8 solution (MedChemExpress, Monmouth Junction, NJ, USA) for 1 h. Once the culture was terminated, the 96-well plate was placed under a microplate reader, and the absorbance of each well at 450 nm wavelength was measured.

### EdU assay

Huh-7 and HepG2 cells were inoculated in the 24-well plate and cultured for 24 h. After that, the cells were incubated with 5 μmol/L EdU medium (Beyotime Biotechnology, Shanghai, China) for 2 h and rinsed with PBS. Next, the cells fixed with paraformaldehyde, and then incubated with 2 mg/mL glycine for 5 min. Moreover, after the cells were washed by PBS on the shaker, the cells were incubated with 0.5% Triton X-100, and incubated for 10 min on the shaker, and then the cells were washed with PBS twice, 5 min each time. Ultimately, the cells were stained with Apollo for 30 min at room temperature and then incubated with DAPI staining solution for 20 min. Subsequent to PBS cleaning, the cells were observed and counted under a fluorescence microscope.

### Transwell assay

Transwell chamber (Costar, Cambridge, MA, USA) was utilized to measure cell migration and invasion. The density of cells suspension was adjusted with serum-free medium to 5 × 10^5^ cells/mL, and 200 μL of cell suspension was subsequently added into the upper compartment of each Transwell chamber, with 500 μL of complete medium loaded into each lower compartment. After 24 h of culture, non-migrating or non-invading cells in the upper compartment were wiped off with cotton swabs. Moreover, the cells passing through the filter were fixed with 95% alcohol and stained with crystal violet dye solution at ambient temperature for 15 min. The number of migrated or invaded cells was counted in 5 visual fields under the inverted microscope, and then the average was calculated, to indicate HCC cells’ ability of migration or invasion. Matrigel (1:10; BD Biosciences, Franklin Lakes, NJ, USA) was adopted for invasion experiment, but not for migration experiment.

### Dual-luciferase reporter gene assay

The wild-type (WT) binding sequence or mutated (MUT) binding sequence between miR-140-3p and circ_0008274 or between miR-140-3p and GRN 3ʹ-UTR was amplified and cloned into pmirGLO Dual-Luciferase miRNA Target Expression Vector (Promega, Madison, WI, USA) to construct the luciferase reporter gene vectors including circ_0008274 WT, GRN WT, circ_0008274 MUT, and GRN MUT. Furthermore, the above vectors and miR-140-3p mimics or its negative control were accordingly co-transfected into 293 T cells, respectively. 48 h later, the luciferase activity was determined by the dual-luciferase reporter gene assay system (Promega, Madison, WI, USA).

### RNA immunoprecipitation (RIP)

RIP assay was performed with Magna RIP RNA binding protein immunoprecipitation kit (Millipore, Billerica, MA, USA) as instructions. The RIP lysis buffer was applied to lyse Huh-7 and HepG2 cells, and 100 μL of whole cell extract was subsequently incubated with RIPA buffer (Beyotime Biotcchnology, Shanghai, China) containing magnetic beads coupled with human anti-Argonaute2 (Ago2) antibody (Millipore, Billerica, MA, USA) at 4°C for 6 h, with a normal mouse IgG (Millipore, Billerica, MA, USA) as a negative control. The aforementioned samples were collected, and washed with washing buffer, and subsequently incubated with proteinase K at 55°C for 30 min to separate RNA-protein complexes from magnetic beads. In addition, the extracted immunoprecipitated RNA was used for qRT-PCR, to detect the enrichment of circ_0008274 and miR-140-3p.

### Western blot

Cells were lysed by RIPA lysis buffer (Beyotime, Shanghai, China), and total cell proteins were extracted after the centrifugation and the concentration of the protein in the supernatant was determined by BCA method. After the loading buffer was added, the supernatant was immediately heated in a water bath at 100°C for 10 min to denature the protein. Subsequently, the protein was separated via SDS-PAGE and subsequently transferred to polyvinylidene fluoride (PVDF) membrane (Millipore, Bedford, MA, USA), which was then blocked with 5% skimmed milk for 1 h at room temperature and then incubated overnight at 4°C with the primary anti-GRN antibody (ab208777, 1: 500, Abcam, Cambridge, UK) and anti-GAPDH antibody (ab9485, 1: 2000, Abcam, Cambridge, UK). Furthermore, rinsed with TBST, the membrane was incubated with Goat Anti-Rabbit IgG H&L (ab205718, 1:1000, Abcam, Cambridge, UK) at ambient temperature for 1 h, and washed by TBST. Eventually, ECL chemiluminescence solution (Beyotime, Shanghai, China) was added on the PVDF membrane, and the protein bands were developed, with GAPDH as the internal reference. Ultimately, the gray values were calculated by ImageJ (NIH, Bethesda, Maryland, USA).

### Statistical analysis

All experiments were replicated three times. SPSS 22.0 (SPSS Inc., Chicago, IL, USA) was adopted for the statistical analysis, with the measurement data expressed as ‘mean ± standard deviation’. *t*-test was employed for comparisons between the two groups, and one-way ANOVA was applied for comparisons among the means of multiple groups; counting data were expressed in a contingency table, and the differences between the two groups were analyzed by χ^2^ test. Statistically, *P* < 0.05 is meaningful.

## Results

We hypothesizes that circ_ 0008274 promotes the progression of HCC through miR-140-3p/GRN axis. In this study, we confirmed that overexpression of circ_0008274 promoted the multiplication, migration, and invasion of HCC cells by CCK-8 assay, Edu assay and Transwell assay. Additionally, with dual-luciferase reporter gene assay, RIP assay and Western blot, we clarified the interactions among circ_0008274, miR-140-3p and GRN.

### Circ_0008274 is in high expression in HCC tissues and cells

Through analyzing circBase database, we learnt that circ_ 0008274 was generated from the transcript of UGGT2 gene (Figure 1(a)). Next, we downloaded GSE97332 dataset from GEO database and analyzed the differentially expressed circRNAs in HCC tissues compared with adjacent tissues. According to the criteria (log_10_|fold change| > 1, *P* < 0.05), we observed that circ_0008274 expression level was markedly increased in HCC tissues relative to adjacent tissues (Figure 1(b,c)). Consistently, qRT-PCR showed that circ_0008274 expression was dramatically higher in HCC tissues than that in adjacent tissues (Figure 1(d)). Additionally, chi-square test proved that the high level of circ_0008274 was associated with larger tumor size and higher TNM stage of the HCC patients (Table 2). Compared with THLE-3 cells, circ_0008274 expression in HCC cell lines (SNU-182, Huh-7, HepG2, SK-Hep1) was significantly up-regulated (Figure 1(e)). In addition, we found that circ_0008274 was dominantly located in the cytoplasm of HCC cells, implying that it could probably function as a competitive endogenous RNA (ceRNA) (Figure 1(f)).

**Table 2.** Correlation between clinicopathological features and expression of circ_0008274 in HCC tissues

Pathological Parameters	Number (n = 55)	Circ_0008274 expression High(n = 28) Low(n = 27)		*P-Value*
Gender				0.3341
Male	38	18	20	
Female	17	10	7	
Age (years)				0.1103
<50	40	23	17	
≥50	15	5	10	
Tumor size (cm)				0.0329*
>5	38	23	15	
<5	17	5	12	
TNM stage				0.0425*
I+ II	23	8	15	
III+IV	32	20	12	
Degree of differentiation				0.0780
Low, medium	33	20	13	
High	22	8	14	

*P < 0.05.

### Circ_0008274 promotes the malignant biological processes of HCC cells

**Figure 2.**
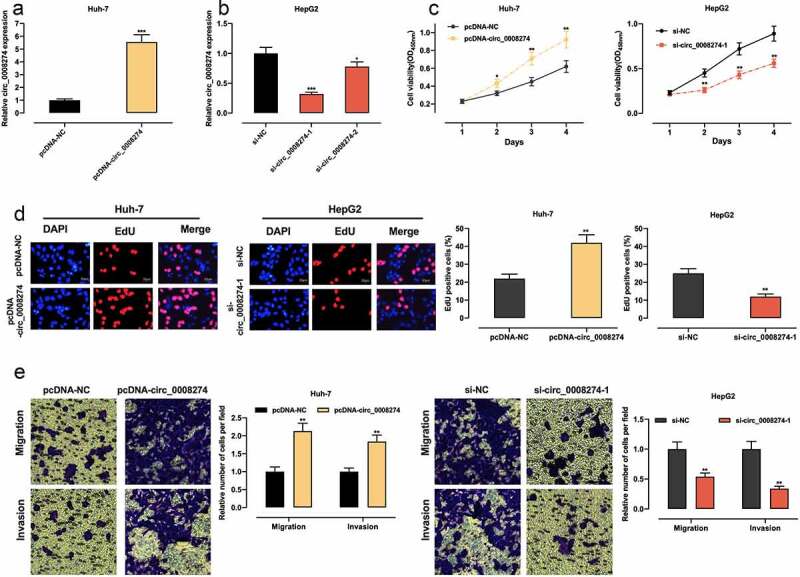
Circ_0008274 promotes the proliferation, migration and invasion of HCC cells

Given that circ_0008274 has the lowest expression in Huh-7 and the highest expression in HepG2 among the four HCC cell lines (Figure 1(d)), we transfected circ_0008274 over-expression plasmid into Huh-7 cells, and transfected si-circ_0008274-1 and si-circ_0008274-2 into HepG2 cells, and the transfection efficiency was verified by qRT-PCR (Figure 2(a,b)). In the subsequent experiments, we chose si-circ_0008274-1 with higher knockdown efficiency. CCK-8, EdU and Transwell assays indicated that, compared to the control group, circ_0008274 overexpression in Huh-7 cells greatly promoted cell viability, migration, and invasion, while circ_0008274 knockdown significantly inhibited cell viability, migration, and invasion of HepG2 cells (Figure 2(c-e)).

A&B. pcDNA-NC or pcDNA-circ_0008274 was transfected into Huh-7 cells, and si-NC, si-circ_0008274-1 and si-circ_0008274-2 were transfected into HepG2 cells. Then the expression of circ_0008274 was detected by qRT-PCR.C&D. CCK-8 and EdU assays were used to detect the effects of overexpression or knockdown of circ_0008274 on the proliferation of Huh-7 or HepG2 cells.E. Transwell assay was used to measure the effects of overexpression or knockdown of circ_0008274 on the migration and invasion of Huh-7 or HepG2 cells.* *P* < 0.05, ** *P* < 0.01, and *** *P* < 0.001.

### Circ_0008274 directly targets miR-140-3p

**Figure 3.**
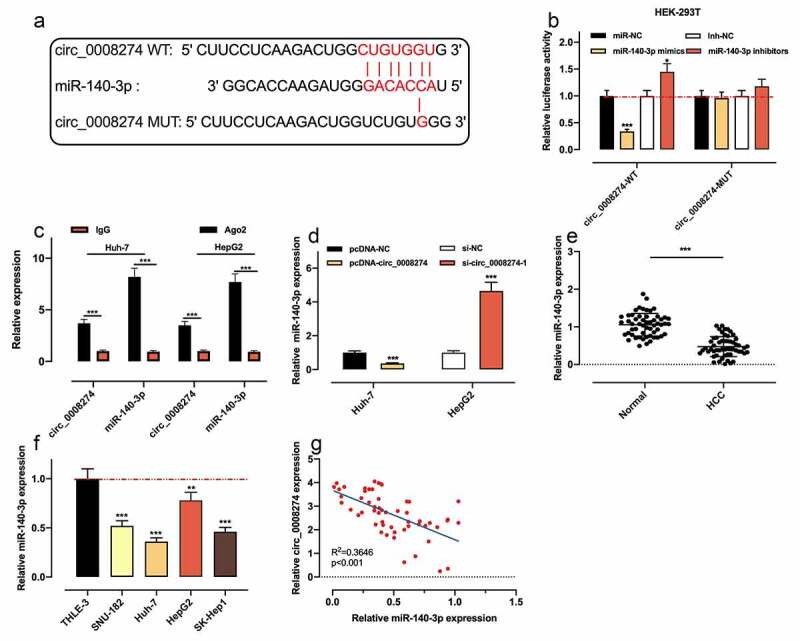
Circ_0008274 adsorbs miR-140-3p

To further clarify the mechanism of circ_0008274, we searched the Circinteractome database and discovered that there was a complementary binding sequence between circ_0008274 and miR-140-3p (Figure 3(a)). Dual-luciferase reporter gene assay indicated that as against the control group, miR-140-3p up-regulation dramatically repressed the luciferase activity of circ_0008274 WT reporter, while miR-140-3p inhibition worked oppositely; whereas that of the circ_0008274 MUT reporter was not significantly affected by miR-140-3p overexpression or inhibition (Figure 3(b)). RIP assay showed that circ_0008274 and miR-140-3p were markedly enriched in Ago2 as against IgG group (Figure 3(c)). qRT-PCR showed that circ_0008274 upregulation remarkably restrained miR-140-3p expression in Huh-7 cells, but circ_0008274 depletion in HepG2 cells functioned oppositely (Figure 3(d)). qRT-PCR also showed that compared with normal adjacent tissues and THLE-3 cells, miR-140-3p expression level in HCC tissues and cell lines was demonstrably declined (Figure 3(e,f)). In addition, Pearson correlation analysis uncovered that circ_0008274 expression was negatively correlated with miR-140-3p expression in HCC tissues (Figure 3(g)). The above results confirmed that miR-140-3p was the downstream target of circ_0008274.

(a).The potential binding site between circ_0008274 and miR-140-3p was predicted by Circinteractome database.(b). The effects of miR-140-3p mimics or miR-140-3p inhibitors on the relative luciferase activity of the reporters carrying the sequence of circ_0008274, were detected by dual-luciferase reporter gene assay.(c). RIP assay was used to detect the enrichment degree of circ_0008274 and miR-140-3p, to indicate their interaction.(d). The expression of miR-140-3p in HCC cells was detected by qRT-PCR after overexpression or knockdown of circ_0008274.(e).The expression of miR-140-3p in HCC tissues and adjacent tissues was detected by qRT-PCR. (f). The expression of miR-140-3p in HCC cell lines and THLE-3 cells was detected by qRT-PCR. (g). Pearson correlation analysis showed the correlation between circ_0008274 and miR-140-3p expressions in HCC tissues.* *P* < 0.05, ** *P* < 0.01, and *** *P* < 0.001.

### Circ_0008274 promotes the progression of HCC via targeting miR-140-3p

**Figure 4.**
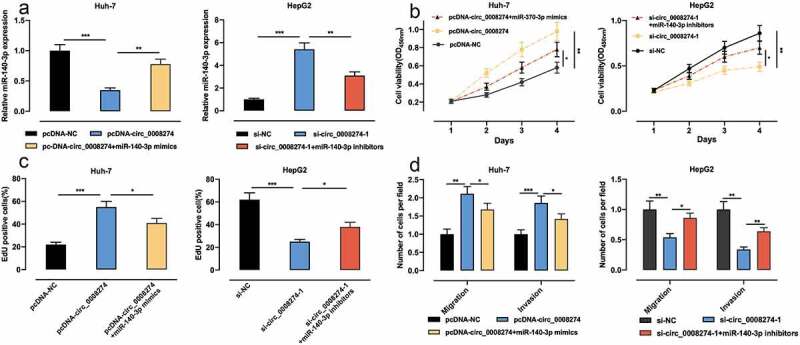
Circ_0008274 can promote the malignant behaviors of HCC cells by targeting miR-140-3p

To study whether circ_0008274 is implicated in regulating the progression of HCC through adsorbing miR-140-3p, we transfected pcDNA-NC, pcDNA-circ_0008274, pcDNA-circ_0008274+ miR-140-3p mimics into Huh-7 cells, and si-NC, si-circ_0008274, si-circ_0008274+ miR-140-3p inhibitors into HepG2 cells, respectively, and qRT-PCR was used to verify that the transfection was successful (Figure 4(a)). Up-regulation of circ_0008274 could promote the proliferation, migration and invasion of Huh-7 cells, while transfection of miR-140-3p mimics weakened these effects (Figure 4(b-d)); knocking down circ_0008274 inhibited the proliferation, migration and invasion of HepG2 cells, but these inhibitory effects were partially counteracted by transfection of miR-140-3p inhibitors (Figure 4(b-d)).

(a).pcDNA-NC, pcDNA-circ_0008274 and pcDNA-circ_0008274+ miR-140-3p mimics were transfected into Huh-7 cells; and si-NC, si-circ_0008274-1 and si-circ_0008274-1+ miR-140-3p inhibitors were transfected into HepG2 cells. MiR-140-3p expression was detected by qRT-PCR.B & C The proliferation of Huh-7 or HepG2 cells were detected by CCK-8 and EdU experiments.D. Transwell assay was used to detect the migration and invasion of Huh-7 or HepG2 cells.* *P* < 0.05, ** *P* < 0.01, and *** *P* < 0.001.

### Circ_0008274 up-regulates CRN expression via repressing miR-140-3p in HCC cells

**Figure 5.**
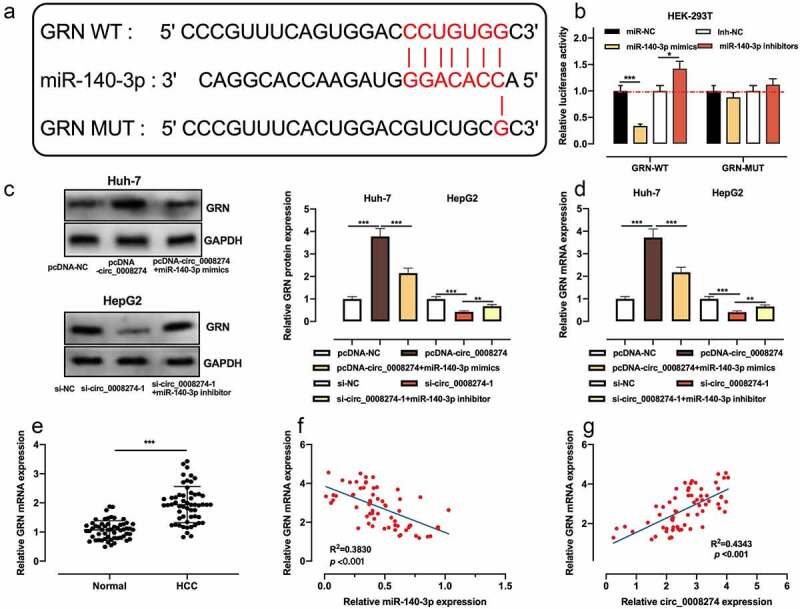
Circ_0008274 regulates miR-140-3p/GRN axis

To further elucidate the downstream mechanism of circ_0008274/miR-140-3p axis in HCC, we searched the Targetscan database and learnt that GRN was one of the downstream targets of miR-140-3p (Figure 5(a)). Dual-luciferase reporter gene assay indicated that up-regulation of miR-140-3p significantly restrained the luciferase activity of GRN WT reporter, while miR-140-3p inhibition functioned oppositely, but that of GRN MUT reporter was not significantly affected by the selectively regulation of miR-140-3p (Figure 5(b)). Next, qRT-PCR and western blot showed that circ_0008274 overexpression increased GRN protein and mRNA expressions in Huh-7 cells, but this effect was weakened by miR-140-3p overexpression; knockdown of circ_0008274 inhibited GRN protein and mRNA expression level in Huh-7 cells, but this effect was weakened by miR-140-3p inhibition (Figure 5(c,d)). Compared with adjacent tissues, GRN mRNA expression in HCC tissues was greatly elevated (Figure 5(e)). The correlation analysis proved that GRN mRNA expression is negatively interrelated with miR-140-3p expression in HCC tissues (Figure 5(f)), but positively with circ_0008274 expression (Figure 5(g)). Collectively, GRN was proved to be the downstream target of miR-140-3p in HCC cells, with its expression negatively modulated by miR-140-3p and positively regulated by circ_0008274.

A.The potential binding sites between GRN mRNA 3’-UTR and miR-140-3p were predicted by TargetScan database.B.The relative luciferase activity was detected by dual-luciferase reporter gene assay.C&D. The effects of circ_0008274 and miR-140-3p on the expression of GRN protein and mRNA were detected by western blot and qRT-PCR.E. The expression of GRN mRNA in HCC tissues and adjacent tissues was detected by qRT-PCR.F&G. Pearson correlation analysis was used to detect correlations between miR-140-3p expression and GRN mRNA expression, and miR-140-3p expression and GRN mRNA expression in HCC samples.* *P* < 0.05, ** *P* < 0.01, and *** *P* < 0.001.

## Discussion

CircRNA was once considered to be the byproduct of aberrant splicing [13]. In recent years, as reported, circRNA is endogenous, stable, abundant and conservative in mammalian cells, and it has tissue and space-time specificity [14,15]. CircRNA is involved in regulating biological processes through many ways, such as decoying miRNAs and blocking its inhibition on target genes, interacting with RNA-binding protein and changing the function of the proteins [16]. The abnormal expression of circRNA is related to the tumorigenesis and progression of HCC. For instance, circ_102034 is elevated in HCC tissues, and in terms of mechanism, circ_102034 promotes NR2F6 transcription by recruiting TIP60 to the promoter region of NR2F6 gene, thus promoting the multiplication and metastasis of HCC cells [17]. In another study, circ_100338 is reported to be up-regulated in HCC tissues, which is related to the poor prognosis of HCC patients; mechanistically, circ_100338 plays a tumor-promoting role via regulating the miR-141-3p/RHEB axis and controlling mTOR signal pathway [18]. Circ_0008274 expression is significantly up-regulated in papillary thyroid carcinoma, which is associated with TNM stage and lymph node metastasis, and circ_0008274 depletion significantly suppresses the multiplication and invasion of thyroid papillary carcinoma cells [9]. Another study reports that, Circ_0008274 is up-regulated in HCC tissues and correlated with poor prognosis in patients with HCC; circ_0008274 silencing suppressed tumor growth *in vivo* and blocked proliferation, colony formation, cell cycle progression, migration, and invasion of HCC cells *in vitro* via miR-526b-5p/RAB1A axis [19]. Consistently, we also found that circ_0008274 was in high expression in HCC tissues, which was associated with the increase of tumor size and TNM stage of the HCC patients. Functionally, circ_0008274 overexpression accelerated the multiplication, migration, and invasion of HCC cells. Our results validated that circ_0008274 was vital in promoting the progression of HCC.

MiRNA, as a kind of non-coding RNA that modulates gene expression in many biological processes, has been linked to the progression of various tumors including HCC [20]. For example, miR-23 c inhibits HCC cell multiplication and induces apoptosis via targeting ERBB2IP [21]. miR-140-3p has been reported to be an important regulator in tumorigenesis [22]. For example, miR-140-3p is a tumor suppressor in lung cancer, which can restrain the multiplication and invasion of lung cancer cells by targeting ATP8A1 [23]; miR-140-3p enhances the sensitivity of HCC cells to sorafenib via targeting pregnenolone X receptor [24]. Reportedly, miR-140-3p is also targeted by circRNA [25]. Circ_NTRK2, for example, accelerates the metastasis of esophageal squamous cell carcinoma via modulating miR-140-3p/NPIP1 axis [26]. In the present work, we observed that miR-140-3p expression is reduced in HCC tissues and cells. We also demonstrated that circ_0008274, mainly located in cytoplasm, sponged miR-140-3p and negatively regulated miR-140-3p expression in HCC. Briefly, we concluded that circ_0008274 promoted HCC progression by targeting miR-140-3p.

GRN, also called progranulin (PGRN), is a protein rich in cysteine and composed of 593 amino acid residues. GRN expression is significantly up-regulated in epithelial cells, immune cells, chondrocytes and neurons with rapid metabolic cycle [27]. In recent years, it is found that GRN is related to many physiological and pathological processes, including early embryonic development, inflammatory response and injury repair [28]. Importantly, there are also many reports about the relationship between GRN and tumor biology [29]. For example, GRN expression is significantly up-regulated in glioma cells, which is associated with the poor prognosis of patients [30]; GRN expression is raised in HCC tissues, which is related to larger tumor size, venous infiltration and early intrahepatic recurrence after the surgery [31]. Besides, GRN-specific neutralizing antibody can repress the vascular endothelial growth factor levels and inhibit angiogenesis in HCC, which indicates that GRN is the potential target for HCC therapy [32]. In the present work, we found that GRN was a target of miR-140-3p, and it could be positively regulated by circ_0008274. Our work suggested that circ_0008274/miR-140-3p axis contributed to the dysregulation of GRN in HCC.

## Conclusion

This study confirms that circ_0008274 up-regulates GRN by competitively inhibiting miR-140-3p expression, thus facilitating the malignant biological behaviors of HCC cells. Our study may offer a new understanding of the pathogenesis of HCC. Nonetheless, in this work, only *in vitro* assays are designed and performed, and *in vivo* experiments are required to further authenticate our demonstrations in the following work. Besides, a larger number of clinical samples is required to evaluate the potential of circ_0008274 as a prognostic biomarker in HCC.

## Supplementary Material

Supplemental Material

## Data Availability

The data used to support the findings of this study are available from the corresponding author upon request.

## Research highlights

What are the new findings?

(1) Circ_0008274 is up-regulated in HCC tissues and correlated with poor clinicopathological parameters.
(2) Circ_0008274 promotes the proliferation, migration and invasion of Huh-7 and HepG2 cells.
(3) Circ_0008274 promotes the progression of HCC by regulating miR-140-3p/GRN axis.

How will our research affect clinical practice in the future?

Circ_0008274/miR-140-3p/GRN axis provides promising diagnosis markers and therapy targets for HCC.

## References

[1] Hartke J, Johnson M, Ghabril M. The diagnosis and treatment of hepatocellular carcinoma. Semin Diagn Pathol. 2017 Mar;34(2):153–159. 28108047 10.1053/j.semdp.2016.12.011

[2] Dhir M, Melin AA, Douaiher J, et al. Update of treatment options and controversies in the management of hepatocellular carcinoma. Ann Surg. 2016 Jun;263(6):1112–1125. 26813914 10.1097/SLA.0000000000001556

[3] Yao JT, Zhao SH, Liu QP, et al. Over-expression of CircRNA_100876 in non-small cell lung cancer and its prognostic value. Pathol Res Pract. 2017 May;213(5):453–456. 28343871 10.1016/j.prp.2017.02.011

[4] Bruix J, Reig M, Evidence-Based Diagnosis SM. Staging, and treatment of patients with hepatocellular carcinoma. Gastroenterology. 2016 Apr;150(4):835–853. 26795574 10.1053/j.gastro.2015.12.041

[5] Qu S, Liu Z, Yang X, et al. The emerging functions and roles of circular RNAs in cancer. Cancer Lett. 2018 Feb;1(414):301–309. 10.1016/j.canlet.2017.11.022 29174799

[6] Yu J, Yang M, Zhou B, et al. CircRNA-104718 acts as competing endogenous RNA and promotes hepatocellular carcinoma progression through microRNA-218-5p/TXNDC5 signaling pathway. Clin Sci (Lond). 2019 Jul 15;133(13):1487–1503. 31278132 10.1042/CS20190394

[7] Li Q, Pan X, Zhu D, et al. RNA MAT2B promotes glycolysis and malignancy of hepatocellular carcinoma through the miR-338-3p/PKM2 axis under hypoxic stress. Hepatology. 2019 Oct;70(4):1298–1316. 31004447 10.1002/hep.30671

[8] Zhou GK, Zhang GY, Yuan ZN, et al. Has_circ_0008274 promotes cell proliferation and invasion involving AMPK/mTOR signaling pathway in papillary thyroid carcinoma. Eur Rev Med Pharmacol Sci. 2018 Dec;22(24):8772–8780. 30575918 10.26355/eurrev_201812_16644

[9] Ito S, Hirabayashi K, Moriishi K, et al. Novel sex-dependent differentially methylated regions are demethylated in adult male mouse livers. Biochem Biophys Res Commun. 2015 Jul 10;462(4):332–338. 25960295 10.1016/j.bbrc.2015.04.137

[10] Chen L, Heikkinen L, Wang C, et al. Trends in the development of miRNA bioinformatics tools. Brief Bioinform. 2019 Sep 27;20(5):1836–1852. 29982332 10.1093/bib/bby054 PMC7414524

[11] Debnath T, Deb Nath NC, Kim EK, et al. Role of phytochemicals in the modulation of miRNA expression in cancer. Food Funct. 2017 Oct 18;8(10):3432–3442. 28782785 10.1039/c7fo00739f

[12] Zhang Q-Y, Men C-J, Ding X-W. Upregulation of microRNA-140-3p inhibits epithelial-mesenchymal transition, invasion, and metastasis of hepatocellular carcinoma through inactivation of the MAPK signaling pathway by targeting GRN. J Cell Biochem. 2019 Sep;120(9):14885–14898. 31044454 10.1002/jcb.28750

[13] Chen LL, Yang L. Regulation of circRNA biogenesis. RNA Biol. 2015;12(4):381–388. 25746834 10.1080/15476286.2015.1020271 PMC4615371

[14] Chang P, Wang F, Li Y. Hsa_circ_0000673 is down-regulated in gastric cancer and inhibits the proliferation and invasion of tumor cells by targetting miR-532-5p. Biosci Rep. 2018 Sep 19;38(5):BSR20180538. 30061181 10.1042/BSR20180538 PMC6146288

[15] Yao Z, Luo J, Hu K, et al. ZKSCAN1 gene and its related circular RNA (circZKSCAN1) both inhibit hepatocellular carcinoma cell growth, migration, and invasion but through different signaling pathways. Mol Oncol. 2017 Apr;11(4):422–437. 28211215 10.1002/1878-0261.12045 PMC5527481

[16] Li R, Jiang J, Shi H, et al. CircRNA: a rising star in gastric cancer. Cell Mol Life Sci. 2020 May;77(9):1661–1680. 31659415 10.1007/s00018-019-03345-5 PMC11104848

[17] Wang L, Long H, Zheng Q, et al. RNA circRHOT1 promotes hepatocellular carcinoma progression by initiation of NR2F6 expression. Mol Cancer. 2019 Jul 19;18(1):119. 31324186 10.1186/s12943-019-1046-7 PMC6639939

[18] Huang XY, Huang ZL, Zhang PB, et al. CircRNA-100338 is associated with mTOR signaling pathway and poor prognosis in hepatocellular carcinoma. Front Oncol. 2019 May 14;9:392. 31157168 10.3389/fonc.2019.00392 PMC6528706

[19] Kong Q, Fan Q, Ma X, et al. CircRNA circUGGT2 contributes to hepatocellular carcinoma development via regulation of the miR-526b-5p/RAB1A axis. Cancer Manag Res. 2020 Oct 15;12:10229–10241. PMID: 33116877; PMCID: PMC7571581. 33116877 10.2147/CMAR.S263985 PMC7571581

[20] Ma J, Zhang F, Sun P. miR-140-3p impedes the proliferation of human cervical cancer cells by targeting RRM2 to induce cell-cycle arrest and early apoptosis. Bioorg Med Chem. 2020 Feb 1;28(3):115283. 31902649 10.1016/j.bmc.2019.115283

[21] Zhang L, Wang Y, Wang L, et al. miR-23c suppresses tumor growth of human hepatocellular carcinoma by attenuating ERBB2IP. Biomed Pharmacother. 2018 Nov;107:424–432. 30103114 10.1016/j.biopha.2018.07.155

[22] Salem O, Erdem N, Jung J, et al. The highly expressed 5ʹisomiR of hsa-miR-140-3p contributes to the tumor-suppressive effects of miR-140 by reducing breast cancer proliferation and migration. BMC Genomics. 2016 Aug 8;17:566. 27502506 10.1186/s12864-016-2869-x PMC4977694

[23] Dong W, Yao C, Teng X, et al. MiR-140-3p suppressed cell growth and invasion by downregulating the expression of ATP8A1 in non-small cell lung cancer. Tumour Biol. 2016 Mar;37(3):2973–2985. 26415732 10.1007/s13277-015-3452-9

[24] Li J, Zhao J, Wang H, et al. MicroRNA-140-3p enhances the sensitivity of hepatocellular carcinoma cells to sorafenib by targeting pregnenolone X receptor. Onco Targets Ther. 2018 Sep 17;11:5885–5894. 30271172 10.2147/OTT.S179509 PMC6149869

[25] Lasda E, Circular PR. RNAs: diversity of form and function. RNA. 2014 Dec;20(12):1829–1842. 25404635 10.1261/rna.047126.114 PMC4238349

[26] Chen X, Jiang J, Zhao Y, et al. RNA circNTRK2 facilitates the progression of esophageal squamous cell carcinoma through up-regulating NRIP1 expression via miR-140-3p. J Exp Clin Cancer Res. 2020 Jul 11;39(1):133. 32653032 10.1186/s13046-020-01640-9 PMC7353745

[27] Wang WX, Wilfred BR, Madathil SK, et al. miR-107 regulates granulin/progranulin with implications for traumatic brain injury and neurodegenerative disease. Am J Pathol. 2010 Jul;177(1):334–345. 20489155 10.2353/ajpath.2010.091202 PMC2893676

[28] Ong CH, Progranulin BA. (granulin-epithelin precursor, PC-cell derived growth factor, acrogranin) in proliferation and tumorigenesis. Histol Histopathol. 2003 Oct;18(4):1275–1288. 12973694 10.14670/HH-18.1275

[29] Jl W, Fu W, Yj D, et al. Progranulin derivative Atsttrin protects against early osteoarthritis in mouse and rat models. Arthritis Res Ther. 2017 Dec 19;19(1):280. 29258611 10.1186/s13075-017-1485-8 PMC5735869

[30] Vachher M, Arora K, Burman A, et al. GRN, and SERPINE1 signature as predictor of disease progression and survival in gliomas. J Cell Biochem. 2020 Apr;121(4):3010–3023. 31710121 10.1002/jcb.29560

[31] Demorrow S. Progranulin: a novel regulator of gastrointestinal cancer progression. Transl Gastrointest Cancer. 2013 Jul;2(3):145–151. 24040621 10.3978/j.issn.2224-4778.2013.02.02 PMC3770304

[32] Ho JC, Ip YC, Cheung ST, et al. Granulin-epithelin precursor as a therapeutic target for hepatocellular carcinoma. Hepatology. 2008 May;47(5):1524–1532. 18393387 10.1002/hep.22191

