# Supplementary figures and images for: Circular RNA circ_0008274 upregulates granulin to promote the progression of hepatocellular carcinoma via sponging microRNA -140-3p
Source: Bioengineered. 2021 May 18;12(1):1890–901. doi: 10.1080/21655979.2021.1926195 (PMC8806606; doi:10.1080/21655979.2021.1926195)

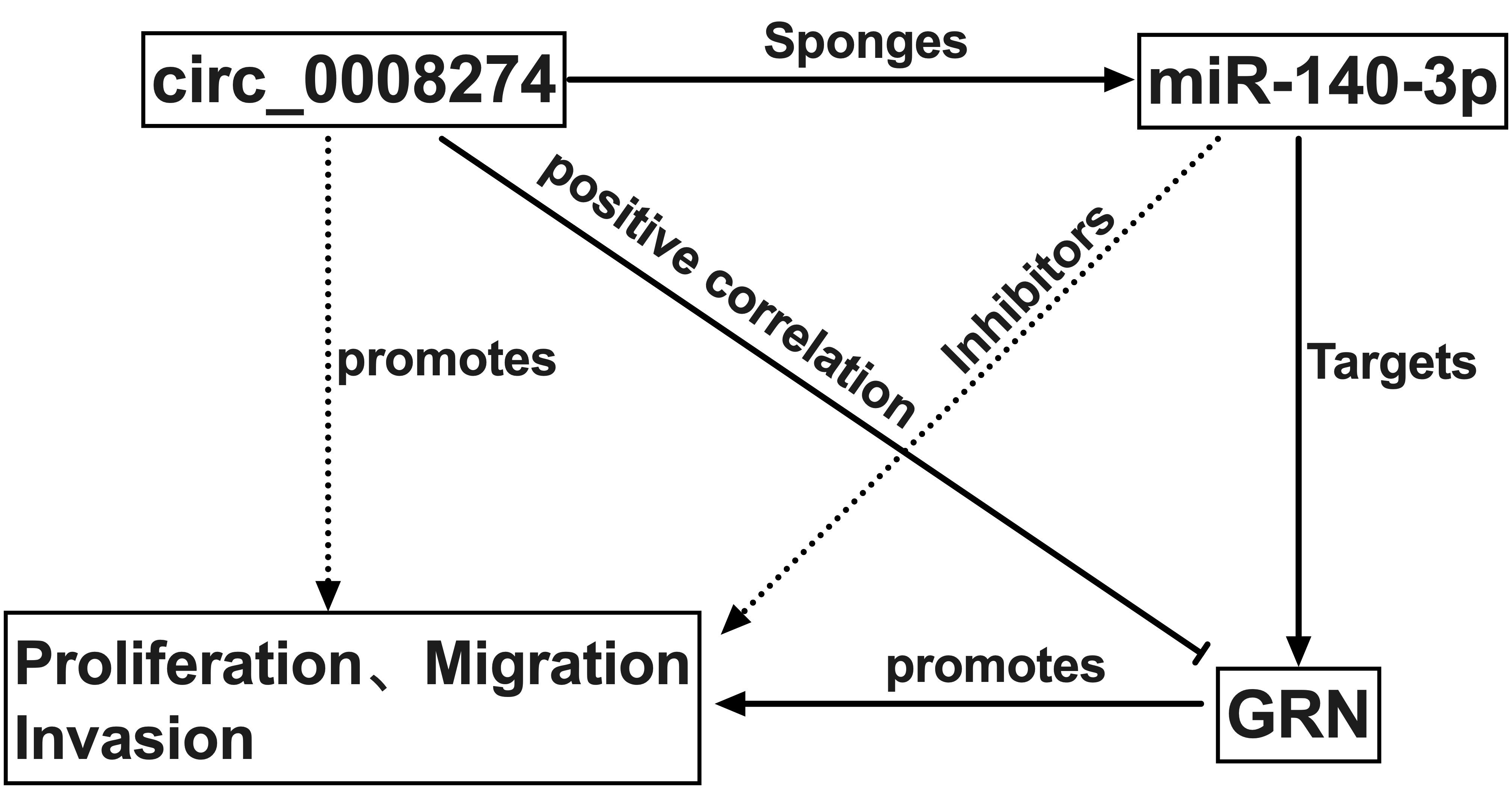

Supplement: Supplemental Material [file KBIE_A_1926195_SM6345.tiff]
